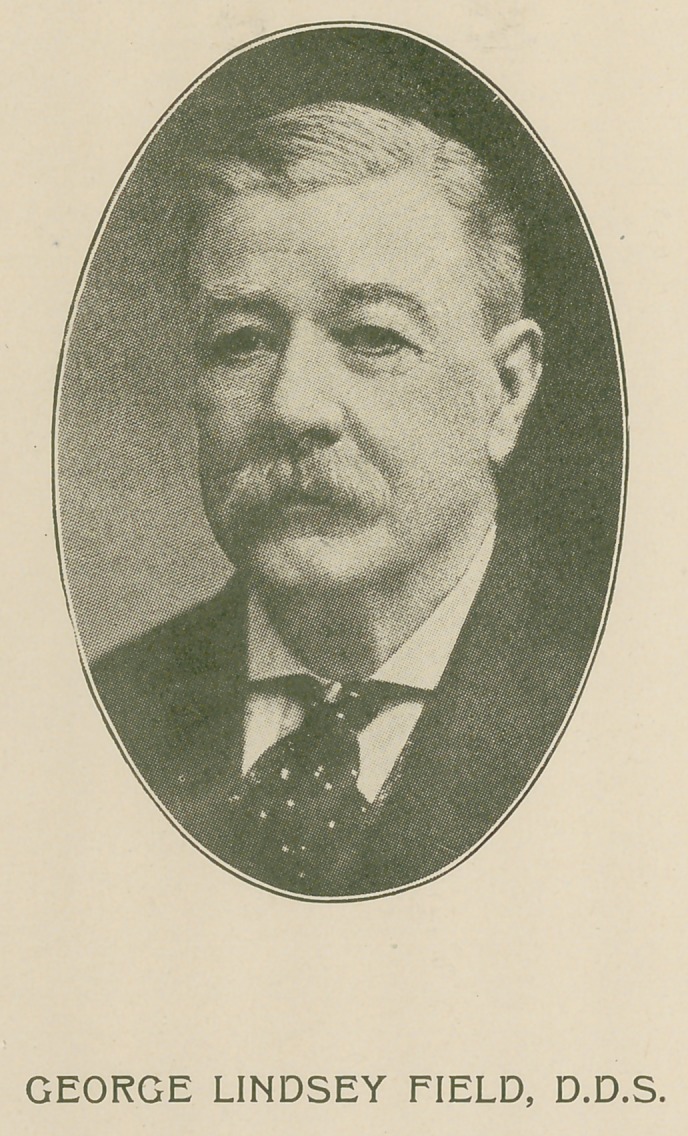# Event and Comment

**Published:** 1907-11-15

**Authors:** 


THE
### DENTAL REGISTER.

Vol. LXI. November 15, 1907. No. 11.

### EVENT AND COMMENT.

### Dr. George Lindsey Field.

On the evening of October 26, 1907, the dentists of Detroit and vicinity gathered at the Fellowcraft Club for a banquet in honor of Dr. Field's fifty years of practice in Detroit. The following is a report of the addresses in part of the evening:

Dr. N. S. Hoff (Toastmaster): Members of the Detroit Dental Society and Friends: It is not necessary for me to spend a great deal of time informing you of the object of this assembly. We are here to celebrate the achievement and victory of an important life; an event of some importance to our honored guest of the evening, and of considerable importance to every man in this room. We have come together not to preach a funeral oration, nor to have a frivolous affair. We have assembled to consider in a somewhat serious, and a friendly way, the life and career of a man whom we all hold dear as a man, and one whom we honor for what he has been and done in his long and devoted professional career. I have no words at my command to express my own feelings to our honored guest to-night. I should prefer to express my sentiments to him in private but I do feel as though we would all like to take his hand and tell him how much we think of him, and what he has been to us personally, and I trust we may have an opportunity to do this at the close of this meeting.

There are many things in Dr. Field's life that are public, and are not so sacred, but we may spend some time talking about them, and your Committee on Arrangements has arranged a program which we will try to follow as nearly as we may that we may cover the essential feature of his career. I feel, for one, in honor bound to pay my respects to our friend as a man who has greatly honored his profession, and, by living a useful life he has also honored himself. Dr. Field, as you know, is the practitioner of longest duration in Detroit at the present time, or has been until his recent retirement from practice. He began the study of dentistry when only sixteen years of age, and he has served the cause long enough to have well earned the retirement which he has taken, and which we hope he may enjoy with all the pleasure that can come to him during the remaining years of his life, which we trust may be many.

Dr. Field began his career at a time when it was not as easy to get into the profession as it now is. We had at that time only two colleges in existence. Dental education was not popular in those days. It was secured by the slow routine practice of serving time in an office, and this Dr. Field did faithfully and truly in the office of a dental practitioner in the city of St.Louis. He spent six years of time in preparing to take up his work in this city; most of this time was spent in the city of St. Louis and the state of Missouri. He came to Detroit at the time when it was a small city of only 40,000 people, and when there were, including himself, only eight practitioners in the city. He has lived a life of usefulness and made a career for himself that we may all envy. When we shall have parcticed for fifty years, I think most of us will feel that we have had all the experience we need, and if we have served our profession as faithfully, we too shall have earned a period of rest. I feel to-night that we have not come here for a funeral oration or anything of that sort, but I wish that we might look upon this occasion as the celebration of a victory, or a triumph, as it were, over the difficulties which our guest has encountered in his career. Living in a period of adjusted professional relations, we do not appreciate the obligations we owe for the courageous stand he has taken in professional matters. It would be difficult for me to enumerate all the good things he has accomplished in his professional career. We can, however, look over the general field and from a hasty review of it see what has been accomplished by the profession in the years he has been in active practice. I hope you will not be wearied as I call to your mind some of the things that he has seen done since he entered the profession.

When he entered the profession there were none of the conveniences that we now have to labor with, very few of the facilities and instruments that we now have. Most of the instruments they had they made themselves and the apparatus and even the furniture in their offices was of the crudest form possible. He has seen, during his life, the system of filling teeth to repair the ravages of decay perfected. The whole system of filling teeth as we know it to-day has been perfected during his life time and within his experience; I mean filling in all the different departments. It was done in those early days in such a crude way that is not so practiced now anywhere. All the modern methods of treatment have come about during his day. Cohesive gold, the greatest advance made in filling materials, was perfected early in his career. and he probably had as much to do with the perfection of this work as any man in our section of the country.

Amalgam and cement fillings, and the various plastic fillings, have all been perfected during his time; certainly none of them, when he began practice, were in anything like a perfect state. Crown and bridge work and modern orthodontia, all are within his memory and experience. Orthodontia, of course, was done before he entered the profession, but in a very crude way indeed. In the prosthetic department he has seen practically the whole field developed and has been personally interested in it to a large extent. He told me that he assisted in making the first continuous gum plate ever made in St. Louis, and he has seen developed the whole process of vulcanite celluloid and cast metal dentures.

In the scientific department he has seen practically the whole range of dental science develop. Previous to his coming into the profession there was no scientific dentistry known of consequence. We knew a great deal in those days about the development of the teeth; some of the old French and English writers studied carefully the development of the teeth, but outside of that very little of scientific value to the profession was known. All dental anesthetics have been developed within his time. All the famous men of dentistry have been developed within his time. The cause of dental caries has been made known. Previous to his time there were three or four theories as to why teeth decayed, but it remained for Prof. Miller to determine what the process of decay was. The whole subject of antiseptic surgery has come within the lives of some of us who have come into the profession more recently than he, and yet it was all within his experience. He has gone through it all and seen it develop up to this present time, and in its present completeness.

The surgery practiced when he entered the profession was empirical, the physician extracted teeth with the turnkey and there were a few who specialized in dental surgery. The whole subject of dental therapeutics has been classified and made scientific within his time. The dentist of his day knew but three or four remedies—creosote, carbolic acid and iodine were the great remedies in his day. They knew nothing better. The entire pathology of the teeth has developed into a more scientific and logical system within his time, exact pathology was known little or not at all at the time he came into the profession. The treatment of the teeth, oral hygiene, oral prophylaxis, are all recent and modern developments, in fact, we have become an educated profession within his day. When he came into the profession there were but two dental colleges in the world: The old

Baltimore Dental College and the Ohio College of Dental, Surgery were the only colleges then in existence. To-day we have fifty-five colleges in the United States, good, bad and indifferent, compared with what there were at the beginning of his professional career. Our literature has grown and developed until to-day we have more than twenty dental journals, and at that time there were only two dental journals in existence. Our literature has become recognized, not only in our own country, but throughout the world. We have the best dental journals published throughout the world. The United States provides the dental literature for the world. During this time we have been writing books; we have been collecting libraries, until to-day there are about twenty libraries, individual and public, varying from 500 to 3,000 volumes. Dr. Field probably has not read all of these books, but he has seen develop the science and art which made them possible. He has seen our profession develop from a mere craft to that of a true and noble profession. When he entered the profession it was usual for a man to invent some method of treatment or practice and conceal it, or to use it only for himself, and never think of giving it to anyone else. If you went into a dental office in those days and a man had anything new that he was doing, you could not get into his laboratory or operating room, and if you got in his laboratory everything would be hidden away out of sight. No secrets were revealed in those days,—it was merely a craft, and not a profession. To-day we have a very different condition of affairs. No man feels that he is doing his duty unless he is learning something that he can give away to somebody else, and this is the motto that every one emblazons on his heart. We have no secrets to-day except by those who handle the commercial part of the profession, and they are not worth considering. Our profession is to-day organized on an ethical basis in every state and territory, and we have several great national organizations. We have dental societies devoted to specialties of dentistry, and we have district, state and local societies.

We have within the state of Michigan nine dental societies. Wherever there are enough ethical dentists practicing in a community they are getting together in societies and social organizations. You will remember a few years ago in the city of Chicago they had a clinic at which there were 3,000 dentists in attendance, and every man was there to learn something new. At our recent national meeting in Minneapolis I am told there were over 1,300 dentists present from all parts of the world. We can claim to be a liberal profession to-day, and we have developed from a mere craft, and done it all within fifty years. It seems almost incredible that we can have made such a tremendous advance as this, and it seems to me that it should be the greatest satisfaction to our honored guest that he has seen it all and has taken his part in this great work for the benefit of humanity. Not only have we this condition of affairs here at home, but dentistry, as you all know, has made a reputation for itself throughout the world. The American dentist is recognized all over the world; wherever there are civilized men there are dentists, and the American dentists are practicing and working in many countries of the world.

Someone has said that we have developed too rapidly, that we have too many dentists and colleges, and dental journals, that we have no use for all these colleges and journals. On the face of it it does seem as though there were more dentists than are needed in the world to-day; but when we come to sift the matter down and look it straight in the face, we find that there are not half enough dentists practicing in the United States who fill teeth as they ought to be filled, and clean them as they should be. We need more, and better dentists, and we regret to loose so good a one from our ranks as our honored guest. We have practicing in the United States, estimated, about 35,000 dentists to-day, and according to our population this means about one dentist to two thousand people. I doubt very much whether one man can take care of five hundred people and do it properly, so that our profession is not over-crowded.

When Dr. Field located in Detroit, he tells me that Detroit was a city of about 40,000 people, and there were but eight dentists here including himself; eight dentists to take care of 40,000 people! Think of it, only one for every five thousand people. But he also tells me that his income for the first year he practiced here was only $150. To-day, I understand there are 400 dentists in Detroit, and 450,000 people—quite a different situation, and I am confident none of Detroit's dentists earns less than a thousand dollars each year. In the next fifty years we shall have twice as many dentists and perhaps twice as many people to take care of.

Dentistry has become so much appreciated in Dr. Field's life time that it has been given a legal standing in every state of the Union, laws protecting the people from the incompetent practitioners have been enacted everywhere, which shows an appreciation of the great value of dental services and the high character required for this service. I think our friend has been most fortunate to have seen all this progress and those of you who are entering the profession to-day, if you see such wonderful advances in the next fifty years, may count yourselves very lucky indeed. Dr. Field said to me to-night, that he wishes he could have had a larger part in this work and could have done more. Those of us who have known him in these past years know that he has had a large part in this work, and as a profession, and as citizens of this community we owe him all honor for his unselfish devotion to our profession. I think it one of the most commendable things the profession of Detroit has ever done to bring this meeting together to-night to celebrate his great achievement and to express our satisfaction with the fates which caused him to cast his lot in this place. He is turning this work over to those of us who are young and able to take up his work. May we have as good a record when we shall be ready to lay down the burden for a period of rest in the sunset of life.

Dr. T. R. Buttrick: Mr. Toastmaster, Dr. Field and

Gentlemen: As the Toastmaster has said, I have had a good deal to do with the arrangement of this program, but you can also thank the committee which so willingly assisted me. Perhaps there is no better expression than to say that all the labor I have bestowed on this has been a labor of love for Dr. Field. I feel that I have no better friend than Dr. Field. I wish that I could put in the best possible language what I feel toward Dr. Field, as my professor, instructor and friend in dentistry. I was fortunate enough to be one of his students in college, and all through my college course he was an inspiration to me. Whenever any difficult problems came up I always went to him and got light. After I graduated I was fortunate enough to have an office in the same building with Dr. Field, and as you all know, a young practitioner has many discouraging things to overcome in his early career, but Dr. Field was always there to encourage me. When difficult problems came to me in my practice I could always get light on the subject from him.

I have a number of letters and telegrams from prominent dentists who could not be here to-night, but who wished in some way to express their great friendship for Dr. Field. Letters were read from Dr. W. B. Tracy, of New York; Dr. J. Ward House, of Grand Rapids, Dr. C. N. Johnson, of Chicago, Dr. B. Holly Smith, of Baltimore, Dr. John A. Watling, of Washington, D. C., and Dr. E. C. Moore, of Detroit, who was too ill to attend the banquet.

The Toastmaster: I feel that we are to be congratulated to-night on having with us a gentleman who has known Dr. Field intimately for many years, and a man who knows our city and its public men, and who has a keen appreciation of the value that these men are to a community. With great pleasure I introduce Mr. G. P. Goodale, a representative journalist of this city, who will speak of Dr. Field as a citizen; with this toast, "Our City, the best in the land; may none who love it be forced to leave it."

Mr. Goodale: When the distinguished British statesman, Joseph Chamberlain, was a guest at a Mayor's dinner in one of the provincial cities of England, and the company were having a capital sense of the enjoyment of the occasion, the host Mayor at one stage of the proceedings leaned over to his guest and said, "Mr. Chamberlain, shall we let them go on enjoying themselves or shall we have your speech now?"

Now, there have been times in my acquaintance with Dr. Field, which has covered a period of exactly forty years—very painful times in my memory—when I had nothing to say; that was subsequent, however, to the invention of the rubber dam. In the napkin days I could express my opinion, but in the rubber dam days he had me where I could not utter the sentences that struggled for utterance. I feel a kind of exultant joy in now having him where I can do the talking and he has to listen.

When I first became acquainted with Dr. Field in a professional way, our acquaintance in the present intimate way began together. He was then housed in the Odd Fellows' Hall, on the west side of Woodward Avenue, between Fort and Congress Streets, in 1867; thence I followed him to the Williams Block, better known as the Kanter Block; again to the Abstract Block, where he was comfortable until my associates and myself, in the proprietorship ofthe *Detroit Free Press*, bought the roof from over his head and turned him out. Then he went up to the Fyfe Building, where, as I said in an article written to celebrate that event fourteen years ago, he had the best front yard of any man in Michigan, and so he has kept it.

The wish that was within me to say the right thing in the right way, and with adequate feeling, and not with mock solemnity or affectation, has led me to doubt the desirability of extemporaneous speech. I have therefor taken the liberty of setting down, in printed words some of the facts that have struggled for utterance since I was asked to speak some words concerning my dear friend and my professional protector.

It shall go hard with me should I fail to testify here and now, and at all other appropriate times and fitting places, my sense of the worth of the kind of manhood and citizenship that is resident in Dr. George L. Field. I am invited to speak of him as a citizen and what I think of him as such. It is little enough that can be formulated in the five minutes at my disposal.

I have known Dr. Field during a continuous period of forty years, and I can testify that he has always seemed to me peculiarly representative of ideal American citizenship. We know him in the family—which is the unit of organized society and the cradle of patriotic impulse—and we know him in the various and many paths along which he has fared from youth to the honored and proud estate of the patriarch. I do not think he has once swerved from the track of conscientious regard for public duty.

It is true that by calendar count his May of life has fallen into the sere and yellow leaf; but there still beats in him the invincible heart of youth, and there still animates him the unconquerable spirit of enterprise. Old Dr. Field is simply unthinkable. It must appear, then, that he has diffused an atmosphere of cheer wherever the hour hath found him. It must also be clear to all his acquaintances that he has been steadily helpful to everybody within the immediate sphere of his personality; and we (some of us anyhow) know that he was always careful to hurt his helpless and dependent friends in that ominous chair of his just as little as may have been thought to comport with professional prerogative and dignity.

I have been looking him over with critical particularity since I received the command to appear here in his behalf. Do you ask what I have found? Well, it were more direct, perhaps, to say that I have not been able to perceive even incipient caries in his character of citizen; and there is no appearance of tartar on his conduct. Added to these significant and normal conditions is the fact that he has kept his civic stomach sweet with the juices of patriotism and pride of city, commonwealth, country and profession. We do not think of him in connection with seekers for political preferment; but the man whose political shibboleth is "measures, not men," might, I think, be trusted with the solemnest obligations that his fellow men could lay on him.

To have acquitted one's self well in the family and in the stated employments of life; and to have earned the esteem of neighbors, friends and community, is to have impressed upon those neighbors, those friends and the body of that community, the signet of good citizenship. These things our friend has done, let us continue to honor him.

Dr. Field, like some others of us, must of course be conscious that the shadows are falling around him. But with his still keen relish of the joys of companionship, and with a duly grateful sense of blessings enjoyed he can smile and say: "Yes, this is my late afternoon of life. Let the calm evening come. That, too, I shall enjoy. And let the darker night fall when it must. I shall be ready. But above all, never let me forget that I walked long in the sunlight."

THE TOASTMASTER: The remark of the last speaker that he could not enter into any fulsome or sentimental praise of Dr. Field seemed to me very pertinent to the occasion. We do not any of us think that Dr. Field was a perfect man, and if we undertook to sense him by a single sense we might find faults in his career, but as we sum up his whole life and look at it in the general, it seems to me that we get a broad view of it and find what a large and valuable man he has been to us, not only in our profession but in this community as well.

We are unfortunate in the fact that Dr. Jackson, who was to have responded to the next toast, "Dr. Field as a Dentist," is unable to be with us to-night, and we learned of it so late that it has been impossible for any one to make preparations to respond to his toast; however, at my earnest solicitation Dr. Bowles has consented to say something upon this topic and I know that he is capable of saying something that will be appreciated. "Dr. Field as a Dentist; may we have his wit to discover the truth and his courage to practice it."

Dr. G. C. Bowles: Mr. Toastmaster, Dr. Field and Gentlemen: As the toastmaster has said, there has been little time for me to prepare to take the toast of Dr. Jackson. I can sit down with one or two of you gentlemen and tell you what I know about Dr. Field so that you will understand me, but in this large presence I seem to be stricken with aphasia, my thoughts leave me. and what thoughts I have are hard to express. I have been thinking of the benefits of the Dental Society and its influence in acquainting us one with another. There we get to know each other's personality and the characteristics of each other. Dr. Field has not been an active member of our Dental Society since I have been a member, so I have not had the privilege of knowing him as intimately as some of you, and of taking him by the hand and learning from that store of information that he has gathered.

Those of us who have seen Dr. Field's work know that he was a master in his profession. Our toastmaster said that dentistry in the earlier days was a craft. Much of it is a craft to-day, and Dr. Field is a Master Craftsman. I have seen gold fillings which were inserted by Dr. Field before the war, and the teeth are to-day sound and in perfect condition. I think if we put in gold fillings as Dr. Field puts them in we would not hear so much about inlays. Dr. Field has always been seeking for the best in dentistry. Someone told me this evening that he was not a scientific, but an eminently practical man; and wherever there was anything practical, Dr. Field sought it out, and believed in giving his patients the very best that was known in dentistry, and he believed in getting fees commensurate to the services rendered. And for this the profession of to-day owes him much.

In speaking with Dr. Field this evening, he was overcome somewhat, as he said: "I received a great many letters congratulating me upon my retirement, but I do not think it is altogether a matter of congratulation." He evidently loves his profession, and I could not extend any sentiment to the profession more appropriate than that. If any of us shall have the pleasure of practicing for fifty years, may we too retire with regrets for having to do it, but let us love our profession to the end. It seems to come to few to leave our profession,—so trying in every way—with regrets, with a sense of dimness of the eyes and pulsation of the heart. It shows to me that there is still a great deal of youth in Dr. Field.

As Dr. Johnson wrote in his letter: "May the sun shine on Dr. Field as long as he lives, and may he live as long as he wants to."

TOASTMASTER: I regret very much to have to announce more trouble: Dr. Hall, who was to respond to the toast, "Dr. Field as an Educator," is necessarily absent this evening on account of illness in his family, which prevented his being here. In view of this fact, I have gained the consent of a gentleman here to speak for us on this topic, also mpromptu, and I trust you will bear with him in what he may have to say:

This trouble reminds me of the old darkey whose husband was ill. Someone said to her: "Aunt Mandy, how is your husband getting along?" "Oh," she says, "I'se discouraged; he war getting along all right, but now the Doctor says he's got the convalescence." We are in hopes that the convalescence we have will be sufficient to satisfy our desires, in what Dr. Graham may have to say in regard to Dr. Field as an Educator. He was one of Dr. Field's pupils and knows him as an Educator and Teacher, and we shall be pleased to hear his tribute to him as such.

DR. DON. M. GRAHAM: Mr. Toastmaster, Dr. Field and Gentlemen: I think after the remarks of the last two gentlemen it would appear sacrilege on my part to afflict you with anything that I might be able to say. The subject is a difficult one to respond to and I am very sorry that Dr.

Hall is unable to be here and respond to this subject, for I know of no person who could do justice to this subject so well as he.

The subject is a broad one, covering Dr. Field's relationship to the students, his relationship to the public and his relationship with the dentists at large. Being one of his students, I assure you that I am proud to have been so fortunate. As freshmen, we used to gaze as we passed by the clinic door, and as we gazed our wonder grew, and, as our senior year came we reached the clinic room with some fear and trembling, and the first order of business was the examination of our case of instruments; and those of you who know Dr. Field, know that he is very exact and autocratic when he comes to examine the students kit of instruments. We all knew our kit was deficient in many ways, and tried to evade his eagle eye. We did so, and congregated about him, and when he called for No. 1, a kit was passed in and examined, and marked O. K. He was more than pleased to find this kit complete; he called for the next one, and this kit was passed out again; he examined it carefully, O. K.'d it, and called for No. 3, whereupon the kit again changed hands and was passed back to the doctor for examination. By manipulating the kit in this way, the 33 members were O. K'd as having complete kits. I want to tell Dr. Field now that there was only one kit of instruments handed in. We were then assigned to chairs and next in order received our patients. I remember well of a youth advancing to Dr. Field, telling him, with his finger in his mouth, that he had a cavity in the left side of his tooth to fill. The Doctor looked rather pained and aggrieved and motioned him to the chair, and, after examining him carefully said, I want to inform you young man that we do not fill temporary bicuspids.

I went to him one day and said I had finished my filling and that it was "good enough." He said: "Young man, the very best you can do is none to good." This impressed me. I can recall many of the serious things he said to us as well as some of the amusing things.

A lady having a decayed tooth went to him and said, "I wish, Doctor, that I had been born without teeth." He said, "Well, madam, most people are born without teeth."

I think we all owe to Dr. Field a debt of gratitude. He started his practice over fifty years ago, and in those days the dentists had a great struggle to gain standards for their profession. You will remember that they were unable to do so. Universities and Medical Colleges refused to recognize us. In desperation our men were compelled to begin a college of their own. The medical profession then looked with scorn upon dentistry as a profession, but what do we see to-day? The other day I was reading an article by Dr. Osler, and he said that there were only three specialties in medicine that had made scientific attainments. Ophthalmology, Dermatology and Dentistry.

I have been so pleased with this testimonial banquet to-night that I hope some such custom will become habitual with us. When any man has practiced ethically, conscientiously and scientifically his profession for forty years, he deserves to be complimented by a banquet, and reminded in some way of the debt of gratitude we owe.

Dr. Field, with a small number of his associates was one of the pioneers, he was one of the charter members of the State Society. He had the honor of being its President for three terms. In looking over the literature of the State organization, I find that he was not only one of the charter members, but one of the prime movers, one of the committee on organization. He had his name signed as a Notary Public to the first organization papers of the State society. In our local society he was ever active and a useful man, so that it would take a long while, and I have no words to convey to you the extent to which Dr. Field has contributed to our profession. I can only say that it is one of the proudest occasions of my life to be at this banquet and to have it said that I am one of the students of the Doctor's.

The Toastmaster: The next toast is, "Dr. Field's influence in the Profession and in Dental Societies," by Dr.

H. K. Lathrop, Jr. He has, as you are perhaps aware, known Dr. Field's career as well as any other man in the state, and can speak intelligently of his work.

Dr. Lathrop: Mr. Toastmaster, Dr. Field and Gentlemen: I wish I was a journalist that I might write all I feel towards Dr. Field. I also wish I was abler and that I could tell you all I know about Dr. Field. I am neither, and therefore almost wish I had a substitute.

It has been my pleasure to know Dr. Field probably as long, or perhaps longer than any one in this room. I don't want you to think for a moment that I claim to be the oldest man in the room, for I do not. I am only a kid, still, perhaps a little more than seventeen years old, which was my age when I began my career as a dentist in the year 1864, forty-three years ago last August, in the offices of Drs. T. A. White and Joseph Lathrop. At that time Detroit was a small city of only about 47,000 inhabitants, and only eight dental offices and a total of ten dentists, Dr. Field being one of them. To be sure he was not a very old man at that time, but has had plenty of time to grow old since then. When Dr. Field closed his office a month or so ago it left no man practicing here that was in practice when I commenced.

Although there are several men practicing in Detroit that are very much older than I am, they were not practicing dentistry in Detroit at that time. I can assure you, gentlemen, that while it is a very great pleasure to look back over the intervening time, it also gives one a sort of a sad, creepy feeling when one thinks how short the time has been and what a short time it will be before the end comes. But the present recollections much more than balance the sad ones. I have no doubt that I could refer to many little incidents that would bring a smile to Dr. Field's face. I have a distinct recollection of being awakened at five o'clock in the morning (at the old International Hotel at Niagara Falls) by a broom straw tickling my nose, the said straw it is needless to say was in the hand of Dr. Field. When

I was sufficiently awake to have lost my anger, I was kindly invited to have a cocktail. I remember many more pleasant incidents of a similar nature but I will not tire you with them for I am fully aware that you all know Dr. Field, and when I have said that it goes without saying that we have had some mighty fine times during these years that have been slipping away. Dr. Field's influence in the profession and in Dental Societies has been very great, and would require a much longer time than is allotted to the speakers for this occasion. I know also that Dr. Field is a very modest man and I am going to let him down easier than if I were speaking behind his back. But I want to say that there have been few men more helpful to young practitioners than he. This was especially his strong point, it certainly was to me, and I have no doubt it has been to others. He was always very willing to show others what he was doing and how he did it. He had another strong point, and that was to listen to what others had to offer, whether it was some new method or some new appliance, it mattered not if the man was young or old, and he did it in a way that always impressed one that he was terribly in earnest and anxious to help others and be helped by others, all for the good of the profession. Dr. Field's influence was very marked in other ways. Of course you know that the conditions were very different thirty or forty years ago from what they are now. The lines were not nearly so distinctly drawn as they are now in regard to ethics. Many a man who holds his head high now has been guilty of unethical methods, but Dr. Field never. It was a thing he could never tolerate. I suppose there is no man in Michigan that was more active in both local and state societies than he, always willing to do his share and more on committees and every where. In the American Dental Association I suppose there never was a man connected with it that was more untiring in his efforts to make a success of it. Life and energy were his predominating qualities and here is hoping that although he has decided to retire from practice, we shall have him in our societies and associations still, and I confidently expect to see his smiling face and hear his cheerful voice in our conferences for many years to come.

THE TOASTMASTER: I am sure there is no one in this presence to-night but will rejoice that we have with us one of our older practitioners, who has kindly consented to respond to the topic, "Dr. Field as one of the Boys." He has always been one of the boys to us, and is one to-day, although he is older in years than most of us. I am very pleased to introduce Dr. James Cleland.

DR. CLELAND: Gentlemen of the Society: I am afraid you will not hear me to-night; my voice is way down in my boots. I was just wondering what I would say to-night, there has been so much said, and so well said. As I told my friend, Mr. Goodale here, he expressed my sentiments in his speech. I remember my first happy meeting with Dr. Field. I think it was in 1874 or '75. There had been a meeting of the American Dental Association at Niagara Falls, and he was very active there and managed to get them to bring their next meeting to Detroit. He had moved out of the old Odd Fellow's Hall into the new office in the Williams Building, and I think I see that office to-day, it was splendid. He had been in the old office so long that he said he was bound to have something nice in the new one. He had a carpet of roses, a beautiful tapestry carpet and the pattern was roses of all kinds, beautiful pink roses, rosebuds and green leaves. He invited all the dentists in the city, at that time, and perhaps there were fifteen or twenty of them, and we had a nice repast there. I forget how many bottles of champagne there were, and a tier or two of Old Crow, and I tell you that when we got through with that, and got a little Crow, we were in pretty good trim, and we got Dr. Field going and he gave us a little account of his history. He told us how after he got through his apprenticeship he had started into business for himself. He thought he would come to Michigan to visit his parents who lived in Detroit. On the way he wanted to visit an old aunt of his. She lived in the great northwest, and he had quite a little difficulty in finding her. He told how he was hindered in his travels by floods and delays of various kinds, and every time he mentioned his aunt's name we had to drink her health. We got him to mention her name many times, and before we got through that night we were all glorious and over all the ills of life victorious. Well, that inoculated a good spirit amongst the dentists of Detroit and it has remained with them ever since. Previous to that there was little visiting with each other; in fact, they kept aloof, but after that there were many visits with each other and we used to have our meetings in each other's offices, and always had something to cheer us—and this was done all through Dr. Field's good, big heart. I know that many times when I was blue I would go up on the way home, seeing the light in his office, and see him, and always got the right hand of fellowship from him, and he would say,

"How are you, Jimmy?"

"Oh, kind of middling."

"Let me see your tongue." He would say, "You're just alive, and that's all."

Well, he would mix up a little "medicine" for me, and a little for himself. We would sit down and have a "crack" and I tell you there were no blue thoughts then in either him or me. We had lots to talk about, and always had a good time. That has lasted for a great many years; we have never had a cross word with each other; we have alwaye been the same, and I hope we shall remain the same until we get the clap of the cold shoulder, which will not be very long now. Dr. Field is old, but he is not so old as I am, so that I can brag of it in that respect, and I think my health has remained good just on account of those little "drops of medicine."

TOASTMASTER: These little experience meetings are good for the soul. I notice we have a gentleman with us who has been associated with Dr. Field for may years in college work, and I want to hear a word from Dr. Shattuck.

Dr. G. S. Shattuck: Mr. Toastmaster, Dr. Field and Gentlemen; I have a message and a token from an old friend of Dr. Field, which I am requested to present to him at this time. Dr. Field, I know not what is in this package, neither do I know what is written in this letter, but I do know that it is the sentiment of a true friendship and admiration of a life-long friend and professional brother. I will say to you, Dr. Field, that the box may contain diamonds, and if it does, the luster of those diamonds will pale the sentiment which this old friend of yours sends to you. I know, Doctor Field, that you will appreciate this token not for its instrinsic worth or value, but as a token of his friendship and the esteem in which he holds you. It is from your old friend Dr. George Roscoe Thomas, Pasadena, California.

I do not suppose there is a man in this room who has been more closely connected with Dr. Field than I have been for the last 15 years. You all know our relationship in the College; we both taught the same work, and I want to say to you gentlemen, that we have never had a hard word in our lives. We always agreed. If any difficulty arose we got together and it was settled satisfactorily at once. Dr Field has been my best friend and my help through all my struggles in the arduous struggle of college work. When Dr. Field left me I felt as if my great staff had gone and I was left on my own support. I shall always have a good feeling towards my friend Dr. Field.

The Toastmaster: I am sure we all appreciate the sentiment which our friend Dr. Shattuck has expressed; it is the sentiment of us all. The next number on our program is the presentation of a loving cup to Dr. Field as an expression which those of you who are gathered here to-night wish to make in some tangible way of the feeling in which you hold him as a friend and brother. I shall call upon Dr. George Burke, to make this presentation.

Dr. G. F. Burke: Mr. Toastmaster, Honored Guest and Gentlemen: There has been assigned to me a very pleasant duty, and I only wish that I had the utterance of some of the gentlemen that I might better form its expression. Personally I am very glad to be present this evening for the purpose of adding my mite in contributing to the worth of our friend and brother. Dr. Field's uniform courtesy, kindly interest and unswerving fidelity to the highest and best interests of our profession have always been an inspiration to me, as I believe that they have been to a great many others in this city. As has already been said here this evening, it is a very fine thing to have spent such a useful and honorable life, and Dr. Field may well feel proud that now, when he is about to start on a period of well earned rest, that he has the hearty support and love of his fellows in the Dental profession, as manifested by this splendid meeting here this evening. We sincerely wish Dr. Field and Mrs. Field all the good things in this life, and trust they may live long to enjoy them.

It gives me very great pleasure, Dr. Field, to present to you, on behalf of your fellow practitioners, this silver Loving Cup, together with this Scroll containing the lis, of names of those present here this evening. Take itt Dr. Field, and as the days and years go by may your spirit be soothed and comforted by the affection that goes with it from each and every one of us.

The Summers may come and the Summers may go,  
    And the Winters may whiten the earth with their snow,  
But let no earnest joy in the Heavens on high  
    Be more sure than this Cup and its cycle of life.

DR. FIELD: Oh! Lord! I never new before what a good fellow I am. This expression from you in my praise is too much for me. I was afraid that I would break down when I heard what you had prepared for me. I cannot tell you how I feel, for you must go through it if you are ever to know what it is. In receiving this beatuiful cup from you, I shall preserve it as long as I stay here with you, and will leave it to my one child in memory of the good father she had, she never knew she had such a good father.

I wish I could express to you, gentlemen, what I feel. I cannot do it. I am perfectly surprised at what you have done for me in honor of the career I am now closing as a dental practitioner in this city. I have been asked to give a little autobiography of my life, which I have prepared in a very unsatisfactory way to myself, and it may be so to you. I think it my duty to do what I can, if it shall add any to your pleasure to give it to you, and it may be possible that I am not such a man as you have made me out to be. I have been a long time wearing the harness of a dentist. I began when I was almost a child, and I have been at it now for fifty-six consecutive years, a little over, and if you like I will read you what little I have prepared, and will not keep you very long, and if I make any mistakes it is because it is in my own hand writing and is hard to read.

I was born in England and came to New York with my father and mother when I was only a few months old. My father removed from New York to Detroit in 1837. Why he brought *me* along I never knew. Probably he thought that I was too young to go it alone, and therefore I might get lost in the shuffle. I have been told that I was a sweet little baby, and all the girls loved to hold me in their laps that they might toy with my golden locks, which curled so tightly over my little head, that it gave me a kind of staring expression, and I am told that they had to put pennies on my eyes to help me to sleep for this same reason. Well, as time rolled on it was thought advisable to send me to school, *why*, I could not just understand, but as I was willing to believe that my parents knew better about such things than I did, I went, and went regularly, except such little times as I would forget about it and go fishing and that always seemed to engender a certain amount of *coolness* to arise between me and my teacher, who would kindly ask me to remain after school hours, that we might visit a little, and talk the matter over; and it generally ended by there not being any coolness on *my* part when I left for home, for my dear teacher would kindly warm me up before we separated. Oh! but it was lovely—for the teaher, not me. I think that those little seeances that we used to have together drove off all inclination to a torpid liver. I think that all this kind of misunderstandings *might* have been avoided if mother had only written me an excuse to have given the teacher to tell him how sick I had been, or my having to go to my grandmothers funeral, or something of that kind you know. But no, she would say: George, "the school will get to be a sort of 'hum-drum' place if you are not there; they want you to kind of keep things a sizzling as it were." So whenever I could spare the time from my fishing days, I would go to school, but I felt all the time that I was being stunted in both my mental and physical growth in these efforts of mine to keep peace in the family.

Well, the wheel of time they tell me never stops, at any rate, mine never did; that is my own *individual* wheel, and I went along with it, until it rolled me into the great city of St. Louis, in the summer of 1850. Here I lay dormant for a whole year, letting my brain get a rest. At the end of that time it was found that I had grown to that size that I and my two brothers could not occupy the same bed without somebody tumbling out every few minutes; so one of us had to get out and find other quarters, and as it was I that slept on the outside and did all the tumbling, it fell to my lot to be the one to leave the home nest.

Now it so happened that Dr. L. W. Spalding, the leading dentist in St. Louis at that time, was in want of a competent man to assist him to carry on his practice. Now the Doctor's family and my fathers family were close friends, and the Doctor had his attention called to the fact that I was an exceptionally bright and capable young man. Notwithstanding the fact that I was just passing through that which my mother called the "contemptable age," he at once proceeded to lay his plans to secure my services, for he could see at once my natural, inborn ability, to attend the door, and mix up moulding sand, to say nothing of that of running errands, and things of that sort, and he at once made an offer to my father, who was my legal adviser and business agent, to pay the sum of $50.00 for my first years services, along with board and lodging; $75.00 for the second and $25.00 additional for each succeeding year until I should arrive at theage of twenty-one years. when I should have reached my majority. He on his part also promised to teach me habits of industry and good morals, but this latter I think he entirely forgot. So on the fifteenth day of September in the year of of our Lord, 1857 I was made a regular *indentured apprentice*; the articles were legally drawn up and duly signed by Dr. Spalding, my father and myself. I had learned to write my name by that time and I immediately went to work to make myself useful, and in about two weeks had made considerable progress, for at about this time a boy, belonging to one of the best families in the city came into the office to have a tooth extracted, and as the Doctor happened to be out, I thought this a favorable opportunity to demonstrate to him that he had made *no* mistake, in selecting me as an assistant. So I got the boy into the chair, telling him that the *other* Doctor was out, and that I would relieve him of his trouble. I found that the tooth was very loose and would come out very easily. I got hold of the first forceps that came to my hand and went at it, but was disgusted when I found that the top of the tooth had come off and that the root was left behind, and then I went at it again. I could feel the root plainly enough, but it seemed so smooth that I could get no grip on it—and the boy began yelling to beat the band and jumped out from the chair; but I locked the door and made him get back in his seat again, and again I went at it, but with no success, and at last let him go home and proceeded to make a charge on the day book of "one tooth pulled, $1.00," quite satisfied that when the Doctor came in that he would begin to think seriously of offering me an interest in the business. It was not long before the boys father came in with his son for the Doctor to take a look at his mouth; and then the Doctor found that it was a *deciduous cuspid* that I had just pushed off and that it was the cusp of the permanent tooth that I had had so much trouble trying to extract and was just ready to erupt. I have often thought since that I would like to see that permanent tooth when it *had* erupted. That was my first *chair* work.

I would say in memory of my preceptor that he was a most excellent teacher. What ever he did, he did well, and he tried to inculcate such principles in his student, as to what extent he succeeded probably the least said the better, but one thing I will say and thatis that when ever I tried to shirk my work I lost time and had to do it all over again.

After I left Dr. Spalding I went with Dr. H. J. McKellops for a few months. There being offered a larger salary I took the superintendency of the laboratory work for a Dr. Timmie, who had three men in his employ in the prosthetic department, all, as well as Dr. Timmie himself, very inferior workmen; but they turned out a great deal of work, chiefly among the poorer classes; it was all metal work in those days, no such thing as rubber plates, celluloid or anything of a plastic nature. Nearly all of the instruments used in a dental office in those days, are those that are never seen now. But just about this time or possibly about a year before I left Dr. Spalding, there was brought to the notice of the dental profession one of the greatest improvements in prosthetic work that had ever been suggested. I refer to continuous gum work. It was presented so perfect in all its details that there has been little or no improvement on it from that day to this, and that was about fifty-five years ago, and I had the privilege of working on the first piece of this kind in St. Louis. Dr. John Allen, of New York City, was the inventor, then the furnaces were very large and heavy, weighing two or three hundred pounds. I refer more particularly to the Roberts furnace, which I for a time used after I came to this city. The furnace that we now use mostly (electric) you can carry in your pocket without inconvenience.

After leaving Dr. Timmie, who had been appointed dentist to the King of Hanover (I hope that the King was a better King than Timmie was a dentist), I went into business with a dentist by the name of Dunham. We had a very nice office in one of the best locations in the city. But Dr. D. sickened and went east for treatment and died, and the office was closed. As I felt that I was too young and inexperienced to run a city office, I started for the north western part of the state. I had no objective place in view. I had become considerably run down in health from the close confinement in a city office, weighing less than 100 pounds. I had some posters printed in blue and red colors reading "Dentistry, Dentistry, Dentistry, Dr. George L. Field, late of St. Louis, etc." This was long before such a thing as dental *ethics* had been brought into existence or even thought of. I had also some circulars, note size, quite modest in their reading, and of which I should not feel ashamed even at this late day. I visited several towns, in some of which I did pretty well, and in others very little, the last one, the town of Huntsville, Randolph Co., the same place that Prof. Vaughn now of the University of Michigan came from, a man of almost world-wide reputation. It was a small place, of some six or seven hundred inhabitants, now a little city of three thousand or more. Here I became acquainted with quite a number of congenial souls, and here I remained for three years, living a "happy go lucky" kind of life. I was always strongly anti-slavery in principal, but, believing that discretion was the better part of valor. I refrained from ever expressing my sentiments, always having a kind of repugnance to riding on a rail, and Missourians in that day were not over particular as to whether the round side, or the sharp edge of the rail, was uppermost, and I always had my preference. In December of 1856 I decided to make a visit to Dubuque where I had relations, and I started for that place, but got side-tracked by a snowstorm at Mendota, Ills., and was compelled to remain there for a time, or go on to Chicago and from there go west by another road, but this one I found out of commission also. So I decided to go on to Detroit, where my fathers family now lived and whom I had not seen in three years. My father at once opposed my return to Missouri, taking the ground that should I continue my roving life there or elsewhere, or even remaining and making a home in some little western village, that I would forever shut off any possibility of ever taking any prominent position in the profession that I had chosen for my lifes work, and if never before, *this night* convinces me of his wisdom. I had no money, and no friends outside of my immediate family in this city, but I decided to acceed to my fathers wishes and advice. He lent me the sum of $60.00 which now would only be considered an advance payment on a dental chair, and with it I rented a back room on Jefferson Avenue just a few doors west of Woodward Avenue with a narrow entrance of not more than three feet. This had been occupied as a printing office. I had the floor washed, the ceiling calcimined, a cheap paper put on the walls, and then proceeded with the fitting up and this consisted of an ingrain carpet at 75 cents per yard. I sewed it up and nailed it down. I did not attempt to match the pattern, for I did not feel that I could afford to do so, but the pattern being small it did not show badly. Then I bought a second hand easy chair for operative work, a small table on which to place the little box of instruments that I had with me; a lounge without springs for one dollar; three or four hard bottom chairs borrowed a severely plain box stove that would take in cord wood cut in two once and unsplit (another saving), one oil cloth covered table for writing materials; got a sign painted for $7.00, hung it out to the breezes, and was ready for work—whenever it should come. I now have that first operating chair in my bed room and money would not buy it. My first year's business gross, was less than $150.00. My rent $8.00 per month or $96.00 per year.

Detroit was at that time a very conservative city of 40,000 inhabitants, including eight dentists, which would allow 5,000 to each dentist. The names of these men were Drs. Farnsworth, Whiting, Benedict, Cahoon, Ashley, Knowlton, Farmer and myself. There was little or no fraternal feeling among dentists in those days, but rather that of antagonism and selfishness, and I am thankful that I have been permitted to live to this day that I might see this feeling almost entirely eliminated from our ranks. My heart at this time is far too full of gratitude for me to properly express to you my friends and brothers, what I would *wish* to say to you in acknowledgment of this most lovely testimonial with which you have honored me to-night, that it is more than I deserve I cannot but feel. In stepping from the ranks as I am now doing it is with feelings of deep regret. Still I shall ever consider myself as one of you, but with every man the time must come when his work must be considered as finished, and that time has come to me. I have received many beautiful letters of *congratulation* from those who have been my friends and patients for many long years, but it would have seemed more proper had they been letters of *condolence*, for never in my life have I been called upon to feel so strong a strain upon my very heart strings as when I sent out my "Notice of Retirement"—and stepped from my operating chair for the *last time*. Hoping that I have not tired you by this rather long address, I extend to you my most earnest and heart felt thanks for the great honor that you have done me to-night. God bless you all.

I would say that I have been a far better man than I ever thought I was. I feel, I think, as Nazby did when he told the dream he had wherein he thought he had died. He said while he was waiting with Peter at the Gate regarding his case he amused himself by reading his obituary on his tombstone. He said his spirit blushed when he read that stone and found what an exemplary man he was. That is the way I feel to-night. I had no idea that it was possible, but I know you are all truthful, and meant every word you said.

I thank you heartily for the Scroll and this beautiful trophy that you have presented to me, which I shall always hold dear. I bid you all Good Night, and may God bless you.

THE TOASTMASTER: I am sure this meeting to-night will not be without its result. Not a word has been spoken or said in commendation of the work of our friend but what all of us would say is true, and he need have no hesitation in believing that we are earnest in what we have said to him. I am glad this meeting has been held in this public way while our friend is with us. It is easy to say pleasant things about friends who have gone over, but it is far better to say those things to our friends while they are with us, so that they may know we appreciate their sincerity and earnestness. It has been a great pleasure for us to be here to-night, and I know that this meeting will be one of our precious memories as long as we live. I hope it may not be the last we shall indulge in, but there are occasions that come now and then where we can honor our associates, and it seems to me that we can do nothing better for our own spiritual and moral welfare than to take advantage of all such occasions. I sympathize a great deal in the pleasure that the little girl expressed to her mother the first time she went to a circus, she had never gone to any public affair before, except to a prayer meeting. She came home perfectly delighted, and said to her mother, "Mother, if you would just once go and see a circus I do not believe you would ever want to go to a prayer meeting again." Now I feel to-night that if we can have just a few such occasions as this sprinkled along through our careers it would be a wonderful help to us, and would bring us closer together, make us more sympathetic and cordial in our relations with each other, and we would have more forbearance for each others faults.

## Figures and Tables

**Figure f1:**